# Complementation between inactive fragments of SssI DNA methyltransferase

**DOI:** 10.1186/1471-2199-13-17

**Published:** 2012-05-30

**Authors:** Krystyna Ślaska-Kiss, Edit Tímár, Antal Kiss

**Affiliations:** 1 Institute of Biochemistry, Biological Research Center of the Hungarian Academy of Sciences, Temesvári krt. 62, 6726, Szeged, Hungary

**Keywords:** SssI DNA methyltransferase, DNA methylation, 5-methylcytosine, Protein fragment complementation, Protein fusion, Zinc finger

## Abstract

**Background:**

Silencing mammalian genes by targeted DNA (cytosine-5) methylation of selected CG sites in the genome would be a powerful technique to analyze epigenomic information and to study the roles of DNA methylation in physiological and pathological states. A promising approach of targeted DNA methylation is based on the ability of split fragments of a monomeric DNA methyltransferase (C5-MTase) to associate and form active enzyme. A few C5-MTases of different specificities have been shown to possess the ability of fragment complementation, but a demonstration of this phenomenon for a C5-MTase, which has CG specificity and thus can be targeted to methylate any CG site, has been lacking. The purpose of this study was to test whether the CG-specific prokaryotic C5-MTase M.SssI shows the phenomenon of fragment complementation.

**Results:**

We show that truncated inactive N-terminal fragments of M.SssI can assemble with truncated inactive C-terminal fragments to form active enzyme in vivo when produced in the same *E. coli* cell. Overlapping and non-overlapping fragments as well as fragments containing short appended foreign sequences had complementation capacity. In optimal combinations C-terminal fragments started between conserved motif VIII and the predicted target recognizing domain of M.SssI. DNA methyltransferase activity in crude extracts of cells with the best complementing fragment pairs was ~ 4 per cent of the activity of cells producing the full length enzyme. Fusions of two N-terminal and two C-terminal fragments to 21.6 kDa zinc finger domains only slightly reduced complementation ability of the fragments.

**Conclusions:**

The CG-specific DNA methyltransferase M.SssI shows the phenomenon of fragment complementation in vivo in *E. coli.* Fusion of the split fragments to six unit zinc finger domains does not substantially interfere with the formation of active enzyme. These observations and the large number of complementing fragment combinations representing a wide range of MTase activity offer the possibility to develop M.SssI into a programmable DNA methyltransferase of high specificity.

## Background

Cytosine C5-methylation plays important roles in several biological phenomena, such as restriction-modification in prokaryotes, genomic imprinting and carcinogenesis in eukaryotes [1]. This reaction is catalyzed by DNA (cytosine-5) methyltransferases (C5-MTase), which transfer a methyl group from S-adenosyl-methionine (AdoMet) to carbon 5 of cytosine in specific DNA sequences [2]. Prokaryotic C5-MTases contain ten conserved sequence motifs and a so-called variable region located between conserved motifs VIII and IX [3]. The conserved motifs are responsible for the general steps of the methyl transfer reaction [4-9], whereas specific sequence recognition is mediated mainly by the variable region [10][11]. Our understanding of the three-dimensional structure of C5-MTases and of their interaction with substrate DNA is mainly based on the X-ray structures of two enzymes: M.HhaI and M.HaeIII. They revealed that both MTases fold in two domains, the large domain encompassing most of the conserved motifs and the small domain containing the variable region and conserved motif IX. The two domains form a cleft where the DNA substrate fits with the major groove facing the small domain and the minor groove facing the large domain. All specific DNA-protein interactions are at the small domain – major groove interface [12,13]. Eukaryotic C5-MTases are larger proteins but the sequence homology they share with prokaryotic C5-MTases and the available biochemical data suggest that they have the same catalytic mechanism [14,15].

Although the vast majority of characterized C5-MTases function as monomers, there are exceptions: M.AquI (CYCGRG) and M.EcoHK31I (YGGCCR) consist of two polypeptides. The larger subunit of M.AquI contains conserved motifs I – VIII and part of the variable region, whereas the smaller subunit contains the distal half of the variable region and conserved motifs IX - X [16,17]. In M.EcoHK31I, the larger subunit encompasses conserved motifs I – VIII, X as well as the predicted target recognition domain (TRD), and only motif IX is located in the smaller polypeptide [18]. The structural plasticity of C5-MTases is also supported by the phenomenon of protein fragment complementation observed with three enzymes: N- and C-terminal inactive fragments of three naturally monomeric C5-MTases (M.BspRI, M.BsuRI and M.HhaI) can assemble to form active MTase if expressed in the same *E. coli* cell, [19,20].

The goal of this work was to test whether M.SssI, which has the same specificity (CG) as the eukaryotic DNA MTases [21] and therefore has special importance as an experimental tool in the study of eukaryotic DNA methylation, is capable of fragment complementation. In higher eukaryotes, DNA methylation occurs at CG dinucleotides (CpG sites) and is associated with gene silencing [22]. Targeted DNA methylation, i.e. selective methylation of predetermined CpG sites in the genome is emerging as a promising technique for selective gene silencing [23-27]. The applicability of targeted methylation as a research tool or as a potential therapeutic approach critically depends on the specificity of targeting, *i. e.* on the difference of methylation between targeted and non-targeted sites. One approach to increase targeting specificity capitalized on the phenomenon of functional complementation between inactive fragments of the MTase. In the first implementation of this technique complementing N- and C-terminal fragments of the HhaI MTase were genetically fused to zinc finger proteins (ZFP) engineered to recognize different nine bp sequences. When the MTase fragment-ZFP fusion proteins were expressed in the same *E. coli* cell, the targeted M.HhaI recognition site, which was flanked by the two closely spaced ZFP binding sites, became methylated, whereas the other M.HhaI recognition sites on the same plasmid stayed unmethylated [28]. Although this strategy is likely to require improvement to suppress the non-targeted background methylation mainly deriving from the reconstitution of the MTase in unbound state [29], it probably remains the most promising approach for achieving the specificity required for using targeted methylation as a reliable research tool [30,31]. However, of the C5-MTases shown to possess the capacity of fragment complementation, only M.HhaI can be used to target CpG sites, and even M.HhaI can methylate only a small subset of CpG sites (1 in 16, those in GCGC context). To be able to target any CpG site, one needs a C5-MTase with CG specificity such as M.SssI.

Here, we report that M.SssI shows the phenomenon of fragment complementation, thus it is, in principle, suitable for the split fragment approach of targeted DNA methylation.

## Results

### Construction of plasmids expressing truncated fragments of M.SssI

**Figure 1:**
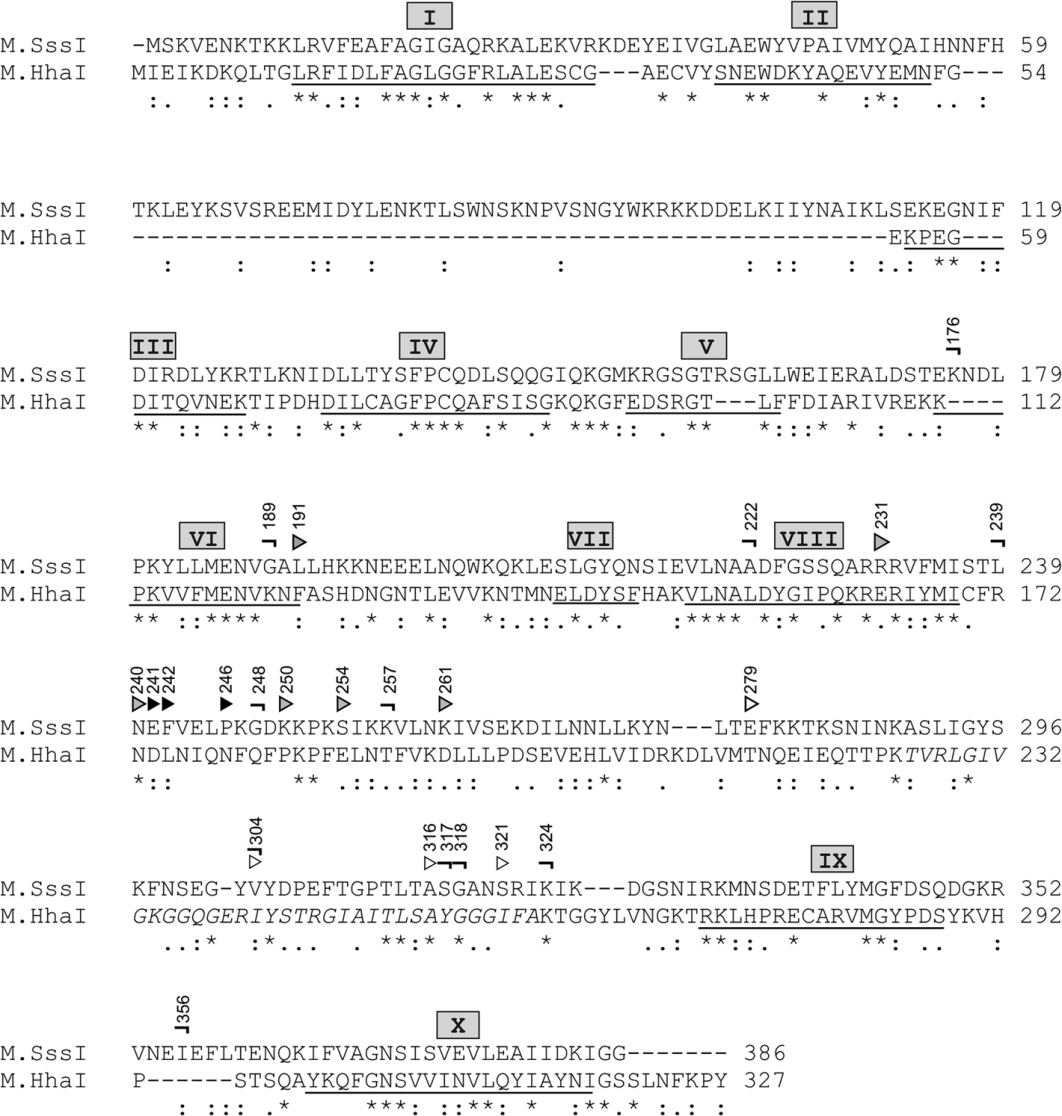
**CLUSTAL W sequence alignment**[33]**between M.SssI and M.HhaI.** Conserved motifs [2,3,8] are underlined and marked by Roman numerals. The TRD of M.HhaI [12] is in italics. The ends of N-terminal fragments constructed in this study are marked by bent symbols and the beginnings of C-terminal fragments by arrowheads above the sequence. C-terminal fragments displaying good, medium or no complementation of the [1–304] fragment are indicated by black, grey and empty arrowheads, respectively.

M.SssI consists of 386 amino acids and contains all conserved sequence motifs typical for C5-MTases [21] (Figure 1). There is no X-ray structure available for M.SssI, but a computational model has been created using previously solved structures of the HhaI and HaeIII MTases [32]. According to this model, M.SssI has the same global architecture as M.HhaI and M.HaeIII, it consists of a large domain comprising conserved motifs I-VIII and X, and a small domain containing conserved motif IX and the TRD. The function of several residues predicted by sequence homology and the computational model was tested by mutational analysis [8].

To test complementation between split fragments of M.SssI in vivo, the gene segments encoding N-terminal fragments were cloned in pBAD24 (Ap^R^), whereas the gene segments coding for C-terminal fragments were cloned in the compatible plasmid vector pOK-BAD (Kn^R^). In both vectors transcription of the target gene is under the control of the *araBAD* promoter and the AraC protein, expression can be induced by arabinose and repressed by glucose [34,35]. Placing the genes of the N- and C-terminal fragments under the same transcriptional control allowed coordinated expression of the two fragments. For future applications, almost all fragments were designed to carry N- or C-terminal 6xHis-tag. Because one of our aims was to explore possibilities of chemical coupling of the MTase to triplex forming oligonucleotides [36], the C-terminal His-tag was engineered to carry a cysteine as the last amino acid (Table 1).

**Table 1:** Plasmids encoding N- or C-terminal fragments of M.SssI

**Plasmid**	**Res.**	**N-terminal extension**^**1**^	**C-terminal extension**^**1**^
pB-Sss[1–189]	Ap		EI
pBNH-Sss[1–176]	Ap	MVPGMH_6_LEC	KLGCFGG
pBNH-Sss[1–222]	Ap	MVPGMH_6_LEC	ASLAVLADERRFSA
pBNH-Sss[1–239]	Ap	MVPGMH_6_LEC	SCFGG
pBNH-Sss[1–248]	Ap	MVPGMH_6_LEC	LAVLADERRFSA
pBNH-Sss[1–257]	Ap	MVPGMH_6_LEC	EAWLFWRMREDFQPDTD
pBNH-Sss[1–304]	Ap	MVPGMH_6_LEC	
pBNH-Sss[1–317]	Ap	MVPGMH_6_LEC	KLGCFGG
pBNH-Sss[1–318]	Ap	MVPGMH_6_LEC	KLGCFGG
pBNH-Sss[1–324]	Ap	MVPGMH_6_LEC	LGCFGG
pBNH-Sss[1–356]	Ap	MVPGMH_6_LEC	KLGCFGG
pB6ZB-Sss[1–239]	Ap	6-ZFP-B zinc finger domain	SCFGG
pB6ZB-Sss[1–304]	Ap	6-ZFP-B zinc finger domain	
pOB-Sss[191–386]	Kn	MVP	SH_6_C
pOB-Sss[231–386]	Kn	MVP	SH_6_C
pOB-Sss[240–386]	Kn	MV	SH_6_C
pOB-Sss[241–386]	Kn	M	SH_6_C
pOB-Sss[242–386]	Kn	MVQ	SH_6_C
pOB-Sss[246–386]	Kn	MV	SH_6_C
pOB-Sss[250–386]	Kn	MVH	SH_6_C
pOB-Sss[254–386]	Kn	MVP	SH_6_C
pOB-Sss[261–386]	Kn	MV	SH_6_C
pOB-Sss[279–386]	Kn	M	SH_6_C
pOB-Sss[304–386]	Kn	M	SH_6_C
pOB-Sss[316–386]	Kn	MVP	SH_6_C
pOB-Sss[321–386]	Kn	MVP	SH_6_C
pSss[191–386]del^2^	Kn	MVP	SH_6_C
pSss[241–386]del^2^	Kn	M	SH_6_C
pOB-Sss[191–386]SS	Kn		SH_6_C
pOB-Sss[241–386]-6ZA	Kn	M	6-ZFP-A zinc finger domain
pOB-Sss[261–386]-6ZA	Kn	MV	6-ZFP-A zinc finger domain
pBS-CAL75	Ap		
pBS-Sss6ZA	Ap		6-ZFP-A zinc finger domain

Numbers in square brackets specify the M.SssI fragment expressed by the plasmid.

^1^ N- and C-terminal extensions were added on purpose (His tag) or are byproducts of the cloning procedure. ^2^Plasmid lacking the *P*_*BAD*_ promoter.

### Functional complementation between inactive fragments of M.SssI in vivo

**Figure 2:**
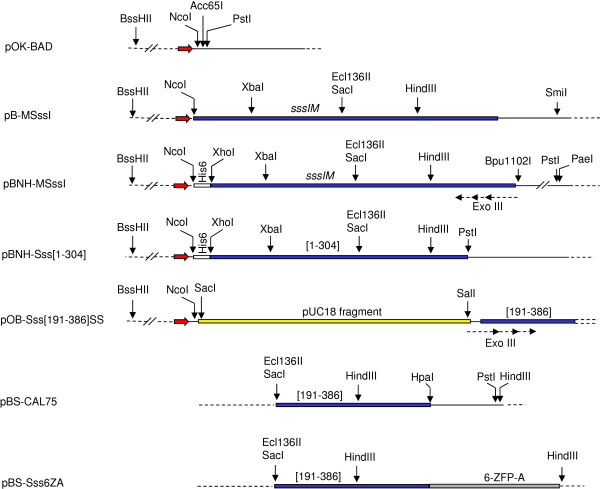
**Schematic map of the relevant regions of some plasmids used in this study.** Blue horizontal bar, *sssIM* gene coding sequence; gray horizontal bar, 6-ZFP-A zinc finger protein gene; red arrow, *araBAD* promoter. Restriction sites used in plasmid constructions are shown. Maps are not drawn to scale.

First two plasmids (pB-Sss[1–189] and pBNH-Sss[1–304]) expressing N-terminal, and four plasmids (pOB-Sss[191–386], pOB-Sss[241–386], pOB-Sss[279–386], pOB-Sss[304–386]) expressing C-terminal polypeptides were constructed. (Figure 2 and Table 1, the numbers in square brackets indicate the first and the last amino acid of the M.SssI fragment encoded by the plasmid.) Some of the break points were selected to approximately match the ends or the beginnings of fragments of the other C5-MTases, for which the phenomenon of fragment complementation has been demonstrated [19][20]. Plasmids coding for N- or C-terminal fragments were introduced individually or pairwise into *E.coli* DH10B. The *recA* host was used in these experiments to minimize the possibility of recombination between homologous segments of the two plasmids. Transformants were grown in the absence or presence of the inducer arabinose overnight. Methylation status of plasmid DNA isolated from the cultures was tested by digestion with the GCGC-specific restriction endonuclease Hin6I whose activity is blocked by M.SssI-specific methylation. The vectors pBAD24 and pOK-BAD contain 32 and 21 Hin6I sites, respectively.

**Figure 3:**
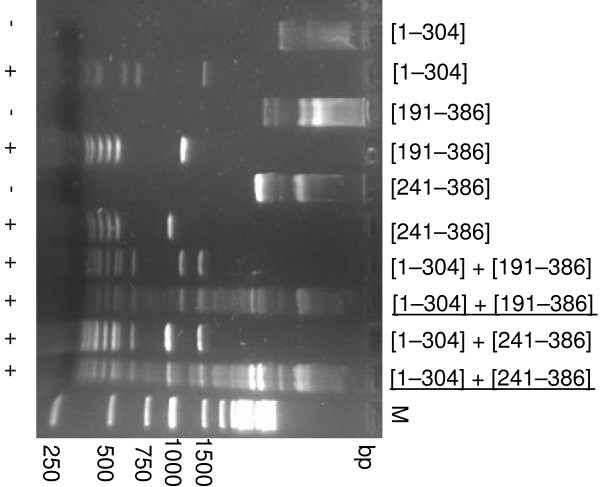
**Functional complementation between inactive fragments of M.SssI.** Hin6I digestion of plasmids expressing the M.SssI fragments shown above the lanes. Protection against Hin6I digestion is a sign of C5-methylation at CG sites. Plasmids indicated by underlining were prepared from cells grown in the presence of 0.5 % arabinose. M, DNA size marker (Fermentas). Undigested and digested samples are identified by – and + signs, respectively below the lanes.

As expected, all plasmids purified from single-transformants were fully digestible with Hin6I, indicating that the truncated fragments were inactive. Similarly, the plasmids purified from double-transformants expressing the fragment pairs [1–189] + [191–386] or [1–304] + [304–386] were completely digestible. However, plasmid preparations purified from the arabinose-induced cultures of the double-transformants containing pBNH-Sss[1–304] plus pOB-Sss[191–386] or pOB-Sss[241–386] were partially protected against Hin6I digestion (Figure 3). This suggested that the N-terminal fragment [1–304] could assemble with the [191–386] or the [241–386] C-terminal fragment to reconstitute the active MTase. The [1–304] + [279–386] combination gave barely detectable protection (not shown). No protection was observed with plasmids purified from uninduced cultures and complementation was more efficient at 30 °C than at 37 °C.

Since the truncated genes of the complementing N- and C-terminal polypeptides contained overlapping segments, it was important to exclude the possibility that MTase activity arose by reconstitution of the intact MTase gene via homologous recombination. To test this, the *araBAD* promoter and part of the *araC* gene was deleted from pOB-Sss[191–386] and pOB-Sss[241–386] leaving the [191–386] and [241–386] coding sequence intact. The resulting plasmids (pSss[191–386]del and pSss[241–386]del) did not complement pBNH-Sss[1–304] (not shown) indicating that for restoration of MTase activity synthesis of the C-terminal peptide was required, and a recombination mechanism can be excluded.

**Figure 4:**
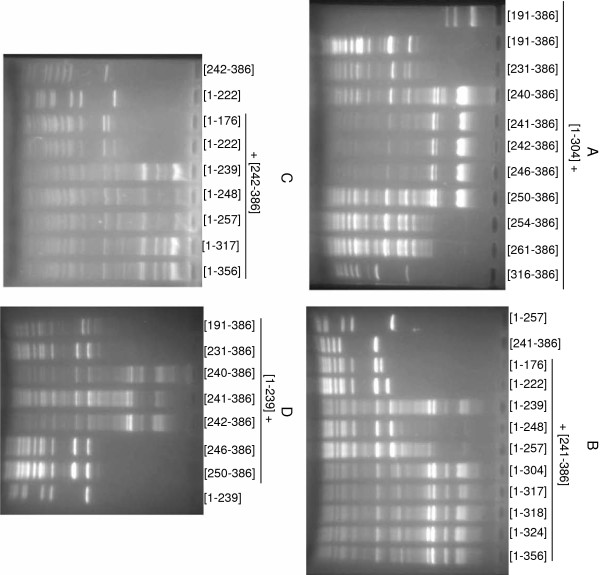
**Dependence of complementation efficiency on fragment length and overlap.** Hin6I digestion of plasmids expressing the M.SssI fragments shown above the lanes (the first sample on panel A was not digested). Plasmids were purified from cultures grown in the presence of 0.05 % arabinose. Protection against Hin6I digestion is a sign of C5-methylation at CG sites.

To determine the optimal C-terminal fragment length for complementation, nested deletions were generated by exonuclease III digestion starting from the N-terminus of the [191–386] fragment as described in Methods. The obtained plasmids encoding the truncated polypeptides [231–386], [240–386], [242–386], [246–386], [250–386], [254–386], [261–386], [316–386] and [321–386] are listed in Table 1. When tested in combination with pBNH-Sss[1–304], some protection against Hin6I digestion was detectable for all fragments having overlaps with fragment [1–304]. The most efficient methylation was observed with fragments starting at 241, 242 and 246 (Figure 4A). These residues are located between conserved motif VIII and the assumed TRD of M.SssI (Figure 1).

In a reciprocal approach, nested deletions were introduced from the 3’-end of the M.SssI gene. Plasmids expressing the following polypeptides were isolated: [1–176], [1–222], [1–239], [1–248], [1–257], [1–317], [1–318], [1–324] and [1–356] (Table 1 and Figure 1). Because in these plasmids the truncated gene was fused to vector sequence, translation termination is determined by the next in-frame stop codon downstream of the PaeI site (Figure 2), and the fragments carry short C-terminal appendages of foreign sequence (Table 1). These plasmids were tested in combination with pOB-Sss[241–386] and pOB-Sss[242–386], two of the plasmids that most efficiently complemented the N-terminal polypeptide [1–304]. The two shortest fragments ([1–176] and [1–222]) did not complement [241–386] (Figure 4B). Complementation by fragment [1–239] was rather efficient, whereas only poor methylation activity was observed with fragments [1–248] and [1–257]. The low complementation capacity of the latter two fragments is probably due to the relatively long foreign sequence at their C-terminus (Table 1). All longer fragments were active in the complementation test and resulted in comparable levels of methylation (Figure 4B). Similar results were obtained when [242–386] was used as C-terminal fragment although in this combination fragments [1-248/257] appeared more active than with [241–386] (Figure 4C vs. 4B). The complementation observed with the [1–239] + [241/242-386] pairs is noteworthy because in these cases there is no overlap between the fragments.

It has been observed in several systems that sequence overlap between the fragment pairs was beneficial or even essential for complementation [20,37,38]. To test this requirement for M.SssI in more detail, a series of C-terminal fragments were tested in combination with [1–239]. Fragments [240/241/242-386] were highly active in pair with [1–239], whereas the few residues shorter [246–386] fragment was already ineffective (Figure 4D). It was shown before that the [1–189] + [191–386] and the [1–304] + [304–386] combinations were inactive (see above). Taking into account that fragments [1–304] and [191–386] were active in combination with fragment(s), which they overlapped, these observations show that the requirement for overlap depends on the location of the break. If the bisection point is in the proximal half of the variable region, no overlap is required, and even the loss of a few amino acids can be compatible with MTase activity. In contrast, if the bisection point is in regions less optimal for fitting the two polypeptides together, a short overlap between the fragment pairs appears to be necessary for the formation of the active complex.

**Figure 5:**
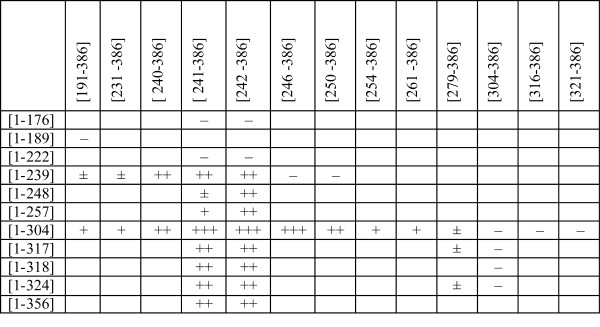
**In vivo complementation between M.SssI fragments.** Some of the fragments carry N- or C-terminal appendages (see Table 1). The approximate level of plasmid methylation as determined by restriction protection assay is indicated by + signs, and the lack of methylation by – sign. Boxes for untested combinations were left empty.

To test how M.SssI tolerates splits in more distal parts of the variable region, plasmids producing different N-terminal fragments were tested in combination with pOB-Sss[279–386] and pOB-Sss[304–386]. Methylation of the plasmid pairs expressing fragments [1-304/317/324] + [279–386] was hardly detectable, and no methylation was detected with the [1-317/318/324] + [304–386] combinations (not shown). These results and the previous observation that the [1–304] + [304–386] pair was inactive (see above) suggest that for efficient complementation the small domain must be intact. A summary of the complementation properties of M.SssI fragments generated and studied in this work is in Figure 5.

To compare M.SssI activities in vivo between representatives of the best complementing fragment pairs and intact M.SssI, extracts were prepared from arabinose induced cells and MTase activity was determined using radioactively labeled AdoMet as described in Methods. The following MTase activities (derived from three independent experiments and expressed in cpm/μg extract protein) were obtained (with the enzyme in parentheses): 1016 ± 369 (full-length), 16 ± 0 ([1–304]), 53 ± 14 ([1–304] + [242–386]) and 65 ± 8 ([1–304] + [246–386]). Assuming similar levels of expression, these values suggest that the activities of even the best complementing pairs is below 5 per cent of that of the intact enzyme.

**Figure 6:**
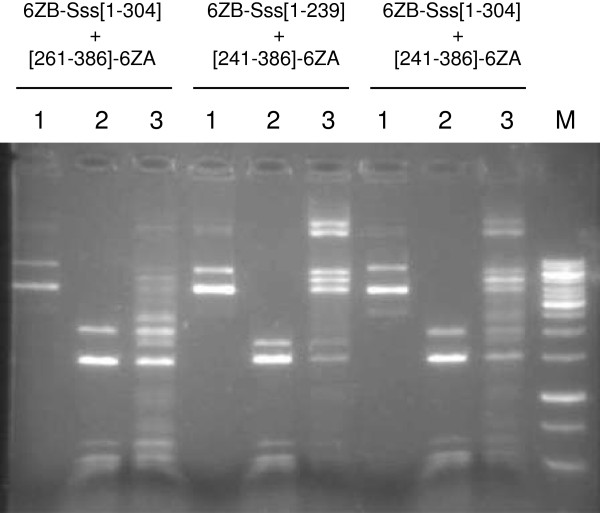
**Complementation between inactive M.SssI fragments fused to zinc finger domains.** DH10B cells contained pB6ZB-Sss[1–304], pB6ZB-Sss[1–239], pOB-Sss[241–386] or pOB-Sss[261–386] in combinations indicated by the names of the encoded fusion proteins above the lanes. Plasmids were purified from cultures grown in the absence or presence of 0.05 % arabinose. Lanes 1, undigested plasmid; lanes 2, uninduced and Hin6I-digested; lanes 3, induced and Hin6I-digested. M, DNA marker.

In the envisaged approach of targeted methylation, targeting is achieved by zinc finger proteins fused to the MTase halves [28]. Statistically, a 16 bp sequence occurs once in the human genome. Thus, for the intended specificity, a 18 bp zinc finger binding site should be sufficiently long as has been demonstrated [39]. Therefore, for testing whether fusion of the M.SssI fragments to zinc finger proteins could impair their complementation ability, we chose six-finger proteins that recognize 18 bp sequences. Two N- and two C-terminal M.SssI fragments ([1–239], [1–304], [241–386] and [261–386]) were genetically fused to the zinc finger domains 6-ZFP-A and 6-ZFP-B described in [40]. The 6-ZFP-B domain was fused to the amino end of the N-terminal fragments, whereas the 6-ZFP-A domain was fused to the carboxy end of the C-terminal fragments to yield pB6ZB-Sss[1–239], pB6ZB-Sss[1–304], pOB-Sss[241–386]-6ZA and pOB-Sss[261–386]-6ZA (Table 1). Fusions to ZFPs slightly decreased complementation capacity in all tested cases (Figure 6 vs. Figure 4), but with the two better combinations substantial activity remained and even for the fragment pair displaying the lowest activity ([1–304] + [261–386], Figure 4A) appending the ZFPs did not fully abolish MTase activity (Figure 6).

## Discussion

In this study we identified N- and C-terminal fragments of the SssI MTase, which can assemble with a counterpart fragment to form active MTase when produced in the same *E. coli* cell. All available evidence supports the conclusion that the observed phenomenon is indeed peptide complementation and not the result of DNA recombination restoring the intact MTase gene. Firstly, deletion of the promoter of two C-terminal fragments abolished complementation (see above). Secondly, plasmid pairs with longer overlaps between the 5’- and 3’ gene segments were not necessarily more efficient in complementation than fragments with shorter overlaps (Figure 4A). Thirdly, in the experiment in which the [191–386] fragment was shortened from the N-terminus by exonuclease III digestion, many of the randomly picked clones contained M.SssI gene segments with substantial overlap with the gene of the [1–304] fragment but fused to the ATG initiation codon in incorrect reading frame. When these plasmids were co-transformed with pBNH-Sss[1–304], methylation was not detectable, confirming that M.SssI activity required synthesis of the C-terminal peptide (not shown). Finally, complementation observed between some plasmids containing non-overlapping gene segments such as [1–239] + [241/242-386] (Figures 4D) also argues against the recombination model.

Although a large number of fragment pairs resulted in detectable M.SssI activity, the most efficient methylation was observed with fragment combinations involving C-terminal fragments starting between conserved motif VIII and the assumed TRD, with a sharp optimum between residues 241 and 246 (Figures 1 and 5). In lack of an X-ray structure it is difficult to rationalize these results but the location of split sites in the region connecting the predicted large and small domains [32] would be consistent with the general principle that fragment complementation is most efficient between folded units [37]. The flexible random coil conformation predicted for the segment Glu241 - Ile262 [32] could also be an advantage for fitting the two subunits together. It is noteworthy that the beginning (K210) of the C-terminal M.HhaI fragment producing complementation was in the same region, between motif VIII and the TRD [20].

Evaluation of the phenotypes of the N-terminal fragments generated by exonuclease III digestion is less straightforward because the extraneous C-terminal peptides appended by the cloning procedure (Table 1) are likely to interfere with assembly of the subunits. In contrast to the C-terminal fragments, the complementation ability of the N-terminal fragments did not show a clear optimum. All overlapping fragments, which did not have longer extraneous C-terminal peptide could complement fragments [241–386] and [242–386] and the complementation capacity of these fragments appeared to be similar (Figures 4 B and C).

This work was motivated by the desire to develop M.SssI into a programmable DNA MTase suitable to methylate unique CpG sites in the genome. Here, as a first step toward this goal, the ability of M.SssI for fragment complementation has been demonstrated and fragment combinations displaying different levels of MTase activity in vivo have been identified. These results open the possibility for developing a system for targetable CpG methylation using the split fragment approach, which will involve construction of fusions between fragments of M.SssI and appropriately designed ZFP targeting domains. The split fragment approach is in many ways reminiscent of the technique using ZFP-FokI chimeric nucleases for targeted DNA cleavage [41]. Low activity of the heterodimers can be an advantage for increasing targeting specificity especially if it derives from the low affinity between the MTase halves [31]. Ideally, the split MTase fragments should not be able to assemble in free state at cellular concentrations but should form active heterodimers due to increased local concentration when co-localized to the target site via the ZFP domains. The best strategy to create a site specific MTase could be to start with fragments, which cannot associate into active MTase as has been suggested [29]. The level of targeting specificity required for methylation of unique sites in the genome will probably need extensive optimization of different parameters governing targetable methylation specificity, such as affinity to target and non-target DNA, affinity between the MTase subunits, distance between the ZFP binding site and the targeted CG, etc.. The large number of complementing fragment combinations representing a wide range of MTase activity will be a rich source of starting material to engineer ZFP-M.SssI fusions for programmable DNA methylation.

## Conclusions

The CG-specific M.SssI, similarly to three other naturally monomeric C5-MTases of different specificity, shows the phenomenon of fragment complementation in vivo in *E. coli.* Fusion of the split fragments to six unit zinc finger domains only slightly decreases the efficiency of complementation. These observations offer the possibility to develop M.SssI into a programmable DNA methyltransferase of high specificity that can be useful in the study of DNA methylation in higher eukaryotes.

## Methods

### Strains and growth conditions

The *Escherichia coli* strain DH10B F^−^*endA1 recA1 galE15 galK16 nupG rpsL ΔlacX74* 80d*lacZ*Δ*M15 araD139* Δ(*ara leu*)*7697 mcrA* Δ(*mrr-hsdRMS-mcrBC*) *relA1 spoT1* λ^−^[42] was used as general cloning host and CJ236 FΔ*(HindIII)::cat* (Tra^+^ Pil^+^ Cam^R^)/*ung-1 relA1 dut-1 thi-1 spoT1 mcrA* as host for site-directed mutagenesis.

Cells were grown in LB medium at 30 or 37 °C. Ampicillin (Ap) and kanamycin (Kn) were used at 100 and 50 μg/ml, respectively. L-arabinose (Sigma) was used at 0.5-0.005 % as indicated at the experiments.

### Plasmids

pBAD24 is an expression vector plasmid, in which transcription of the target gene is tightly controlled by the *araBAD* promoter and the AraC protein [34]. pOK-BAD, a Kn-resistant derivative of pBAD24, has p15A replicon, thus is compatible with ColE1-based plasmids such as pBAD24 [35].

All variants of the *sssIM* gene described in this paper are derived from the allele in the plasmid pCAL7 obtained from New England Biolabs. The *sssIM* allele in pCAL7, considered as the WT allele for this work, carries mutations that change four TGA stop codons, which normally specify Trp in the native host *Spiroplasma sp.*, to TGG (W. Jack, personal communication). The pBAD24-based plasmids pB-MSssI and pBNH-MSssI encode the WT and an N-terminally His-tagged M.SssI variant, respectively (Figure 2).

Plasmids encoding N-terminal M.SssI fragments are based on the vector pBAD24, and have names starting with pB, whereas plasmids that encode C-terminal M.SssI fragments, are based on the vector pOK-BAD and carry names starting with pOB- (Table 1).

Plasmid pB-Sss[1–189] was constructed by deleting the Ecl136II – SmiI fragment of pB-MSssI (Figure 2). Ecl136II cuts after the codons of the conserved E_186_NV motif and SmiI after the stop codon. This deletion created a reading frame ending two triplets downstream of the Gly_189_ codon. Thus, the MTase fragment encoded by pB-Sss[1–189] carries a two amino acid C-terminal appendage ENVGEI (underlined).

pBNH-Sss[1–304] is a derivative of pBNH-MSssI, and encodes the [1–304] fragment of M.SssI. To construct pBNH-Sss[1–304], the M.SssI gene segment corresponding to the peptide A_190_ – V_304_ was PCR-amplified using the primers AK183 and AK184. AK183 contains the Ecl136II (SacI) site located in the middle of the SssI gene. AK184 introduced a stop codon and a PstI site after the triplet corresponding to Val_304_ (Table 2). The PCR-product was T/A-cloned in pTZ57R/T (Fermentas), then excised by SacI–PstI double digestion and transferred into SacI-PstI digested pBNH-MSssI to yield pBNH-Sss[1–304] (Figure 2).

**Table 2:** Deoxyoligonucleotides used in this work

**Name**	**Sequence**	**Properties**^**1**^	**Use**
AK183	GAGCTCTTCTTCACAAGAAGA	M.SssI gene sense strand, positions 566-586. Starts with a SacI site	Forward primer for [190-304]
AK184	GCTGCAGTTAAACATAACCTTCTGAATT	M.SssI gene anti-sense strand, positions 912-895, stop codon and PstI site added	Reverse primer for [1-304]
AK185	GCCATGGAATTTAAAAAAACAAAATCA	M.SssI gene sense strand, positions 835-855, NcoI site added	Forward primer for [279-386]
AK186	GCTGCAGTTAGCAGTGATGGTG	M.SssI gene anti-sense strand, stop codon and PstI site added	Reverse primer for His_6_-Cys-tagged C-terminal fragments
AK188	GCCATGGTTTATGATCCTGAATTTACA	M.SssI gene sense strand, positions 910-930, NcoI site added	Forward primer for [304-386]
AK189	GCCATGGAATTTGTTGAACTACCAAAG	M.SssI gene sense strand, positions 721-741, NcoI site added	Forward primer for [241-386]
AK258	GGCACGCTGACTAAAGGATTTACCGCACTCGGG	6-ZFP-A gene antisense strand	Mutagenic primer for elimination of a HindIII site
AK259	GCAGCTGTTTCTCCAAAGAAGAAGAGAAAAGT		Forward primer for amplification of the 6-ZFP-A gene
AK260	GCTGCAGTTAACCCAGCTCGCCG		Reverse primer for amplification of the 6-ZFP-A gene

Sequences are written in 5’ to 3’ direction. Sequences, which do not correspond to the template DNA, are underlined. ^1^Nucleotide positions are calculated relative to the first nucleotide of the ATG start codon of the WT M.SssI gene.

Plasmids pBNH-Sss[1–176], pBNH-Sss[1–222], pBNH-Sss[1–239], pBNH-Sss[1–248], pBNH-Sss[1–257], pBNH-Sss[1–317], pBNH-Sss[1–318], pBNH-Sss[1–324] and pBNH-Sss[1–356] are deletion derivatives of pBNH-MSssI and were generated by exonuclease III digestion. pBNH-MSssI was digested with Bpu1102I and PaeI, which have single sites in the plasmid downstream of the M.SssI gene (Figure 2). Unidirectional deletions starting from the Bpu1102I end (5’-overhang) were made by exonuclease III [43]. The 3’-overhang of the PaeI end protected the downstream sequences. The M.SssI fragments expressed from the plasmids obtained by exonuclease digestion carry short C-terminal appendages (Table 1) determined by the fused vector sequence downstream of the PaeI site.

Plasmids pOB-Sss[191–386], pOB-Sss[241–386], pOB-Sss[279–386] and pOB-Sss[304–386] are based on the expression vector pOK-BAD and encode C-terminal M.SssI fragments. They were constructed either by direct fragment transfer or PCR amplification from a plasmid expressing a C-terminally His-tagged M.SssI variant. This variant and the derived C-terminal polypeptides used in this work carry a cysteine at the end of the His-tag: KIGGSHHHHHHC. (The C-terminal cysteine, not relevant to the present study, was added for future applications.) Plasmid pOB-Sss[191–386] was constructed by cloning the Ecl136II-PstI fragment encoding the His-tagged [191–386] polypeptide between the filled-in Acc65I site and the PstI site in the pOK-BAD polylinker. The variant encoded by pOB-Sss[191–386] has the following N-terminal sequence MVPL_191_LHKK. The underlined three amino acid extension is encoded by the pOK-BAD polylinker. To construct plasmids expressing the [241–386], [279–386] and [304–386] polypeptides, the respective gene segments were PCR-amplified using AK189, AK185 or AK188 as forward primer and AK186 as reverse primer (Table 2). In pOK-BAD the NcoI site overlaps the translational start codon. To facilitate cloning of the synthesized fragments in pOK-BAD, the forward primers were designed to carry a 5’-extension with an NcoI site. The amplified fragments were inserted between the NcoI and PstI sites of pOK-BAD.

Plasmids pSss[191–386]del and pSss[241–386]del were made by deleting the BssHII–NcoI fragment of pOB-Sss[191–386] and pOB-Sss[241–386], respectively. BssHII cuts in the middle of the *araC* gene and the NcoI site contains the ATG start codon of the truncated MTase genes (Figure 2). Thus, this deletion removes part of the *araC* gene along with the *araBAD* promoter and translational initiation signals for the [191–386] and [241–386] polypeptides.

Plasmids pOB-Sss[231–386], pOB-Sss[240–386], pOB-Sss[242–386], pOB-Sss[246–386], pOB-Sss[250–386], pOB-Sss[254–386], pOB-Sss[261–386], pOB-Sss[316–386] and pOB-Sss[321–386] are deletion derivatives of pOB-Sss[191–386] and were obtained by exonuclease III digestion. To have appropriate restriction sites for unidirectional exonuclease III treatment, pUC18 linearized with KpnI was inserted into the unique KpnI site of pOB-Sss[191–386] located in the gene section coding for the N-terminal MVP appendage (see above) i.e. between the start codon and the gene segment encoding the [191–386] fragment. A plasmid, in which the orientation of pUC18 was such that the unique SacI site was upstream (towards the translational start codon), whereas the unique SalI site was downstream, was chosen for further work. Part of the inserted pUC18, which was not needed for controlling the exonuclease III digestion, was deleted by ApaLI digestion and circularization of the large fragment to yield pOB-Sss[191–386]SS (Figure 2). To generate random 5’-deletions in the gene of the [191–386] fragment, pOB-Sss[191–386]SS was cut with SalI (5’-overhang) and SacI (3’-overhang) and treated with exonuclease III as described above. The SalI end allowed exonuclease III digestion to start whereas the SacI end protected upstream sequences, such as the P_BAD_ promoter and the initiation codon. As byproducts of the cloning steps, some of the M.SssI fragments encoded by these plasmids carry short N-terminal extensions (Table 1).

The plasmids pcDNA3.1mnhk-up1 and pcDNA3.1mnhk-up2 encode the zinc finger proteins 6-ZFP-A and 6-ZFP-B, respectively [40], and were kindly provided by M. Rots. To construct fusions between the N-terminal M.SssI fragments [1–304], [1–239] and 6-ZFP-B, the 6-ZFP-B coding region was transferred, on an NcoI-XhoI fragment, from pcDNA3.1mnhk-up2, into pBNH-Sss[1–304] and pBNH-Sss[1–239] to yield pB6ZB-Sss[1–304] and pB6ZB-Sss[1–239], respectively. For fusions to C-terminal M.SssI fragments, the HindIII site in the 6-ZFP-A zinc finger gene was eliminated by site directed mutagenesis using the mutagenic primer AK258. The resulting 6-ZFP-A gene carrying the silent mutation was PCR-amplified using primers AK259 and AK260 containing PvuII and PstI sites as 5’-extensions, respectively (Table 2). The PCR product was T/A-cloned in pTZ57R/T, then excised by PvuII - PstI double-digestion and cloned between the HpaI and PstI sites of pBS-CAL75, a plasmid containing the 3’-half of the *sssIM* gene with an engineered HpaI site overlapping the M.SssI stop codon (Figure 2). Ligation of the PvuII end of the PCR fragment to the HpaI end of pBS-CAL75 creates in-frame fusion between the MTase and the 6-ZFP-A zinc finger domain (Figure 2, plasmid pBS-Sss6ZA). C-terminal fusions between the M.SssI fragments [241–386]/[261–386] and 6-ZFP-A were constructed by inserting the HindIII fragment of pBS-Sss6ZA containing the 3’-end of the M.SssI gene and the coding sequence of 6-ZFP-A (Figure 2) into the unique HindIII site located in the 3’ half of the M.SssI coding sequence (Figure 2) in pOB-Sss[241–386] and pOB-Sss[261–386] to yield pOB-Sss[241–386]-6ZA and pOB-Sss[261–386]-6ZA, respectively.

Nucleotide sequences of the plasmids are available upon request.

### DNA techniques

Restriction digestion, polymerase chain reaction, agarose gel electrophoresis and cloning in *E. coli* plasmid vectors was carried out using standard procedures [43]. Oligonucleotides were purchased from IDT or were synthesized in this institute and are listed in Table 2. Restriction endonucleases, DNA polymerase large (Klenow) fragment, Taq DNA polymerase, exonuclease III, S1 nuclease and T4 DNA ligase were purchased from Fermentas or New England Biolabs. Site-directed mutagenesis was performed by the Kunkel method [44]. Unidirectional deletions were generated by the combined use of exonuclease III, S1 nuclease, Klenow DNA polymerase and T4 DNA ligase [43]. DNA sequence was determined by an automated sequencer (ABI).

### Methyltransferase assay in crude extracts

*E. coli* cells with plasmids encoding M.SssI fragment(s) or the full-length enzyme were grown at 37 °C to OD_550_ ~ 0.5, then 0.1 % arabinose was added and culturing was continued for 3 hrs at 30 °C. Cells sedimented from 200 ml culture by centrifugation were resuspended in 0.2 ml 50 mM Tris–HCl pH 8.0, 10 mM 2-mercaptoethanol and disrupted by sonication. After removing cell debris by centrifugation, M.SssI activity was determined in 30 μl reactions containing 0.9 μg lambda phage DNA (*dam*^-^*dcm*^-^), 50 mM Tris–HCl pH 8.5, 50 mM NaCl, 10 mM EDTA, 5 mM DTT, 5 μM [methyl-^3^ H]AdoMet and 2 μl cell extract. The [methyl-^3^ H]AdoMet used in the reaction was prepared by diluting the 370 GBq/mmol preparation (NET155, New England Nuclear) to 116 GBq/mmol with unlabeled AdoMet (New England Biolabs). After incubation at 30 °C for 20 min, reactions were stopped by 1 % SDS, then the incorporated radioactivity was determined as described [45]. Protein concentration was determined by the Bradford reaction (Sigma). MTase activity was calculated by dividing the cpm values by the amount of total protein in 2 μl cell extract (in μg) used in the assay.

## Competing interests

The authors declare that they have no competing interests.

## Authors’ contributions

KS-K carried out most of the experimental work and participated in drafting the manuscript. ET constructed some of the plasmids and studied complementation between the plasmid pairs. AK designed the study, participated in the experimental work and wrote the manuscript. All authors read and approved the final manuscript.
